# Cardiovascular Disease Modeling Using Patient-Specific Induced Pluripotent Stem Cells

**DOI:** 10.3390/ijms160818894

**Published:** 2015-08-12

**Authors:** Atsushi Tanaka, Shinsuke Yuasa, Koichi Node, Keiichi Fukuda

**Affiliations:** 1 Department of Cardiovascular Medicine, Saga University, 5-1-1 Nabeshima, Saga 849-8501, Japan; E-Mails: tanakaa2@cc.saga-u.ac.jp (A.T.); node@cc.saga-u.ac.jp (K.N.); 2 Department of Cardiology, Keio University School of Medicine, 35 Shinanomachi Shinjuku-ku, Tokyo 160-8582, Japan; E-Mail: kfukuda@a2.keio.jp

**Keywords:** iPSCs, disease modeling, cardiovascular disease

## Abstract

The generation of induced pluripotent stem cells (iPSCs) has opened up a new scientific frontier in medicine. This technology has made it possible to obtain pluripotent stem cells from individuals with genetic disorders. Because iPSCs carry the identical genetic anomalies related to those disorders, iPSCs are an ideal platform for medical research. The pathophysiological cellular phenotypes of genetically heritable heart diseases such as arrhythmias and cardiomyopathies, have been modeled on cell culture dishes using disease-specific iPSC-derived cardiomyocytes. These model systems can potentially provide new insights into disease mechanisms and drug discoveries. This review focuses on recent progress in cardiovascular disease modeling using iPSCs, and discusses problems and future perspectives concerning their use.

## 1. Introduction

Embryonic stem cells (ESCs) are one of the most promising models for medical research. However, there are several concerns regarding the generation and usage of ESCs. For instance, ESCs are derived from early embryos, and hence the use of human ESC is ethically concerning. Furthermore, it is not possible to generate ESCs from a patient’s own tissues with standard methods, and thus allogenic ESC-based regenerative therapies require immunosuppressants. Therefore, easy methods to generate autologous ESCs from patients’ cells are highly desired. In 2006, Takahashi and Yamanaka established the first induced pluripotent stem cells (iPSCs) by retrovirus-mediated transduction of four specific transcription factors (*Oct3/4*, *Sox2*, *c-Myc*, and *Klf4* also known as the Yamanaka factors) into terminally differentiated murine fibroblasts [1]. The nature of these murine-derived iPSCs was found to be identical to that of ESCs. Shortly thereafter, human somatic cells were successfully reprogrammed into iPSCs, and various techniques and applications for iPSCs had been developed within a few years [2,3,4,5,6,7,8,9]. These considerable innovations have opened up new frontiers in medical science in many respects. As iPSCs possess definite characteristics of pluripotent stem cells, including infinite self-renewal and multipotency, they are expected to be used in a wide variety of applications such as in cell replacement regenerative therapies, developmental biology research, disease modeling, and drug screening [10,11,12]. Although less than 10 years have passed since iPSCs were first generated, iPSC research now spans the globe in a wide range of fields.

One of the most exciting iPSC research areas is disease modeling, in which iPSCs are generated from patients with genetic disorders, namely, disease- or patient-specific iPSCs (PS-iPSCs) [13,14,15,16]. To elucidate disease mechanisms, diseased cells isolated from patients are often examined, but many types of somatic cells, such as neural cells and cardiomyocytes, are difficult to obtain. The differentiation of PS-iPSCs into disease-relevant cells provides researchers with a stable and renewable alternative source of target cells for disease modeling, thus leading to the establishment of this promising field. To date, a number of studies on various diseases have demonstrated that PS-iPSC model systems can recapitulate disease phenotypes similar to those exhibited in actual patients. These systems could help improve our understanding of disease mechanisms, and potentially lead to new therapeutic strategies. In this review, we summarize the recent progress in disease modeling using PS-iPSC systems, particularly in the cardiovascular field, and discuss the problems and future perspectives in this exciting arena.

## 2. Generation of iPSCs and Differentiation into Cardiomyocytes

The original method for iPSCs generation used retrovirus-mediated forced expression of defined transcription factors in murine fibroblasts; however, vast improvements have been established. For example, whereas conventional methods required skin biopsies to obtain dermal fibroblasts, methodological improvements have now shown that iPSCs can be generated from various types of somatic cells, such as keratinocytes or peripherally circulating T cells [7,8,17,18,19]. Moreover, the retroviral to delivery of the Yamanaka factors into somatic cells has the potential risk of random integration of the vector-encoded genes into the host genome, which can result in changes in the expression of endogenous genes and unforeseen mutations. To overcome such problems, transgene insertion-free methods using non-integrating viruses, episomal plasmid vectors, synthetic modified mRNAs, or recombinant proteins have been developed, which have reduced concerns for retrovirus-mediated aberrant genetic changes in iPSCs [20,21,22,23,24,25,26,27]. In spite of accumulating studies, the nature of reprogramming itself remains elusive, and the precise differences between iPSCs and ESCs remains unclear [28], and hence such points are beyond the scope of this review.

To advance the use of iPSCs in the cardiovascular field to applicable research tools and clinical agents, protocols for the differentiation of iPSCs to cardiomyocytes have also been improved [29,30]. It is well known that ESCs can spontaneously differentiate and give rise to all cells of the body, including cardiomyocytes, from the three germ layers [31,32,33,34]. ESCs are derived from early embryos and mimic normal early embryonic development. There have been many attempts to regulate ESC differentiation by various factors, including Wnt, activin A, and bone morphogenetic protein (BMP), which have yielded efficient production of cardiomyocytes [35,36,37,38]. Since the basic properties and differentiation potential of iPSCs resemble those of ESCs, protocols to differentiate iPSCs to cardiomyocytes are based on prior ESC studies. A common method to differentiate ESCs and iPSCs utilizes embryoid body (EB) forming floating culture systems. EBs spontaneously differentiate into all three germ layer-derived cells, including those of the cardiac lineage [31]. To improve the efficiency of cardiomyocyte differentiation from iPSCs, culture conditions have been optimized with various cytokines and growth factors. Kattmann *et al.* demonstrated that stage-specific administration and regulation of key signaling molecules, such as BMP4 and activin/Nodal, during differentiation of ESCs and iPSCs increased the efficiency of cardiac mesoderm differentiation [39,40,41]. Additionally, monolayer culture protocols on Matrigel with defined media have been used to efficiently differentiate cardiomyocytes from ESCs and iPSCs [36,42,43]. Since there are several non-standardized methods of inducing ESC and iPSC differentiation into cardiomyocytes, efforts to further enhance the efficiency, stability, and reproducibility of current methods are underway.

## 3. Characterization of iPSC-Cardiomyocytes

To apply iPSC-derived cardiomyocytes (iPSC-cardiomyocytes) to basic research and medicine, such as employing them in disease modeling, drug screening, and regenerative medicine, it is necessary to first elucidate their physiological properties. Initial studies demonstrated that iPSC-cardiomyocytes displayed normal structures such as sarcomeric organization and gap junctions, which are comparable to those of ESC-derived cardiomyocytes. Electrophysiological analyses showed that iPSC-cardiomyocytes had proper electrophysiological functions, and respond to adrenergic/cholinergic stimulation as well as chemicals that can act on various types of ion channels [44,45,46,47]. In addition, calcium imaging using a Ca^2+^ sensitive fluorescence dye and confocal microscopy revealed that iPSC-cardiomyocytes generated adequate Ca^2+^ transients required for excitation-contraction coupling [48,49,50]. Moreover, these physiological properties of iPSC-cardiomyocytes were comparable among iPSCs derived from various types of somatic cells such as dermal fibroblasts and T lymphocytes [51]. Although there are still several hurdles to overcome, which will be discussed later, these studies indicate that iPSC-cardiomyocytes have the potential for applications in disease modeling, drug testing, and future clinical use.

## 4. Disease Modeling Using Patient-Specific Induced Pluripotent Stem Cells (PS-iPSCs)

### 4.1. Disease Selection 

Disease modeling with PS-iPSCs has garnered great attention, as the biologic characteristics of iPSCs have matched the scientific needs for such cells in medical research. Although some of the mechanisms underlying genetic disorders have now been elucidated through analyses of genetically engineered mice, mouse models do not fully represent human disease phenotypes. In terms of cardiovascular disease research, mice show many differences from humans, for example, with respect to heart size, heart rate, electrophysiological properties, and gene expression patterns. Additionally, analysis of human cells from diseased tissue is helpful in understanding disease mechanisms, but it is extremely difficult to obtain these cells (e.g., neural cells, cardiomyctes) in routine clinical settings. Thus, iPSCs have a strong potential for use in cardiovascular disease modeling.

Before choosing to model a disease with iPSCs, the particular type of human disease should be considered. It is extremely important to select a disease to model with iPSCs based on the biological characteristic of the iPSC system. For instance, the time of disease onset and disease progression through time should be taken into account. In general, younger-age onset diseases are favorable for iPSC disease modeling because most iPSC-derived cells have immature phenotypes and epigenetic markers in cardiomyocytes are known to be different between younger and older hearts [52]. It can also be difficult to model complex diseases that are caused by environmental factors and epigenetic modifications at a later age. Recent research using PS-iPSCs from patients with type 2 diabetic mellitus showed that cardiac phenotypes could be modeled by bringing *in vitro* culture conditions closer to *in vivo* conditions [53]. For disease modeling, it is also important whether target cells can be efficiently and reliably differentiated from iPSCs. Although techniques for differentiation of iPSCs into various cells types have improved, there are still several issues. For example, the remaining epigenetic markers from the original somatic cells might affect differentiation propensity, and each iPSC line has variable differentiation efficiency [54,55,56]. Furthermore, *in vitro* analyses currently allow for the examination of cellular phenotypes but not whole tissues or organs. This limits the *in vitro* study of some diseases whose phenotypes are only fully manifested at the tissue or organ level. Moreover, it is still difficult to form tissues or organs with iPSCs, and hence it is highly challenging to model those diseases with iPSCs.

Taken together, these prior investigations suggested that monogenic disorders with early-age onset and phenotypes that can be represented with iPSC-derived cells would be ideal targets for iPSC disease modeling. Indeed, most studies to date have focused on hereditary disorders that satisfy these conditions (see below).

### 4.2. How to Use PS-iPSC System

**Figure 1:**
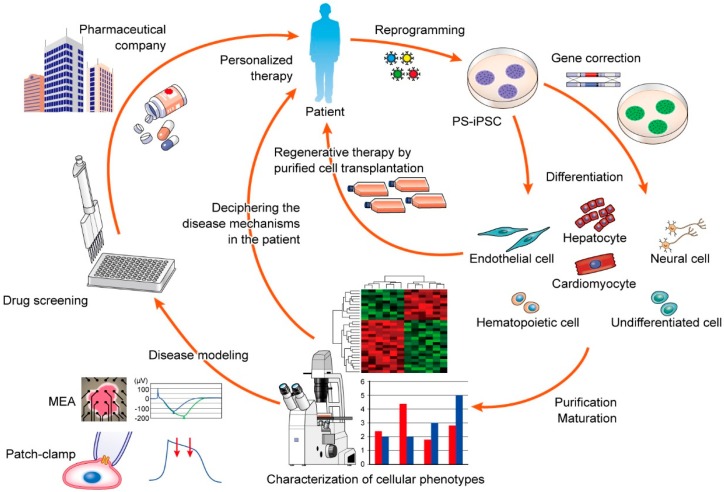
Flowchart of potential applications of patient-specific induced pluripotent stem cell (PS-iPSC) systems. Somatic cells derived from patients with genetic disorders are reprogrammed into a pluripotent state, that is, iPSCs, via induction of defined transcription factors. Subsequently, disease-relevant or mutation-corrected cells are differentiated from iPSCs by gene targeting techniques. Purified and expanded cells are potentially utilized in cellular transplantations. Conversely, differentiated cells can be applied to *in vitro* analyses such as disease modeling and drug testing. In disease modeling, cellular phenotypes are characterized through various experimental methods, potentially providing novel clues to the underlying disease mechanisms, which may further lead to the development of therapeutic strategies. Based on the cellular characteristics, candidate chemical compounds can be evaluated for drug efficacy and toxicity. In the future, PS-iPSC systems could be a useful platform in personalized medicine and efficient drug discovery in collaboration with the drug-manufacturing industry. MEA, multi-electrode array; PS-iPSC, patient specific-induced pluripotent stem cell.

PS-iPSCs have great potential in medical research such as in drug screening, personalized medicine, and regenerative medicine (Figure 1). There are several primary elements in the use of PS-iPSC systems. The first is the generation of iPSCs from patients with a genetic disorder. To elucidate disease mechanisms in PS-iPSC-derived cells, control-iPSCs are also needed. However, the most appropriate “control” remains controversial. In initial studies, iPSCs from unrelated healthy volunteers were often used as controls, but it was difficult to determine whether the observed phenotypes were the direct result of causal mutations because there were many differences in the genetic backgrounds between unrelated individuals. Related but unaffected family members were then used as controls, but there were still many genetic differences. Therefore, isogenic-control iPSCs in which the mutated gene has been repaired by gene-targeting can more accurately depict non-diseased cellular phenotypes [57,58,59]. The second element is to differentiate PS-iPSCs into disease-relevant cells. The third element relates to the use of iPSCs in a clinical setting, including their use in cell replacement therapy. This requires adequate numbers of target cells with high purity to avoid tumorigenesis associated with remaining stem cells. Cell replacement therapy clinical trials using iPSC-derived cells including the transplantation of retinal pigment epithelial cells, neural cells, and cardiomyocytes are now ongoing in animals and humans worldwide [60,61,62,63,64,65,66]. The fourth element relates to iPSC use in medical research such as, in disease modeling and drug screening. This also requires high cellular purity and cellular maturation in conditions that closely reflect *in vivo* conditions. Through disease modeling, novel insights into disease mechanisms can be uncovered, leading to the development of new therapeutic strategies. As the fifth and final element, it is expected that the benefits of personalized medicine can be realized with PS-iPSCs. In a clinical setting, it is not possible to predict a drug’s efficacy or side effects on an individual basis. While some patients may respond well to a drug, others may not. Additionally, some patients exhibit serious side effects whereas others do not. Therefore, *in vitro* analyses with PS-iPSC-derived cells may contribute to the prediction of drug efficacy and side effects in a personalized fashion. In this regard, pharmaceutical companies have already recognized that iPSC-cardiomyocytes can provide a high-quality platform for drug toxicity screening [67,68,69,70,71]. Many new drugs have unexpected cardiac toxicity resulting in sudden cardiac death, which can lead to decisions to abort drug development. Although there are screening systems in place to predict a drug’s side effects on the heart, many drugs that pass initial screening still cause serious side effects. Therefore, improvements in screening methods are needed. PS-iPSC-cardiomyocytes are human cardiomyocytes, which could be used for toxicity screening on an individual basis, thus avoiding concerns related to genetic variation.

To date, disease modeling using PS-iPSCs has been reported for a diverse range of diseases, including neurological degenerative diseases [72,73,74,75,76,77], muscular disorder [78], metabolic diseases [79,80], hematologic diseases [81,82,83], renal disease [84], mitochondrial diseases [85,86], chromosomal disorders [87,88], storage disease [89,90,91], progeroid syndromes [92,93], psychiatric disorder [94], and cardiovascular diseases, which will be discussed more later. These previous reports cover a broad range of diseases that are not only simple monogenic disorders but also multifactorial late-onset diseases. In addition to the common diseases, disease modeling using PS-iPSC systems can potentially be used to study rare diseases where detailed pathological features have yet to be unveiled.

## 5. Cardiovascular Disease Modeling

Animal model research has played a crucial role in deciphering pathophysiological mechanisms in hereditary cardiovascular diseases. However, there are limitations to using animal models for human diseases, which limits the understanding of the underlying mechanism associated with human cardiovascular diseases [95]. Therefore, the focus has shifted to iPSC disease modeling shortly after their development. Table S1 provides a summary of published studies in cardiovascular disease modeling with PS-iPSCs [53,58,59,90,96,97,98,99,100,101,102,103,104,105,106,107,108,109,110,111,112,113,114,115,116,117,118,119,120,121,122,123,124,125,126,127,128,129,130,131]. Most of these studies have focused on hereditary arrhythmia-related channelopathies or cardiomyopathies, and the cellular phenotypes associated with these diseases were evaluated using cardiomyocytes differentiated from PS-iPSCs. The following section is a review of the major results and clinical perspectives from these previous published reports.

### 5.1. Disease Modeling in Channelopathy

Long QT syndrome (LQT) is characterized by prolonged QT intervals on patient electrocardiograms, and are caused by gene mutations in ion channels involved in the repolarization process. Patients with LQT are at higher risk for sudden cardiac death due to fatal ventricular arrhythmias. At least twelve subtypes of LQT syndrome have been identified, and to date, PS-iPSCs have been used to model LQT syndrome types 1, 2, 3, and 8.

LQT syndrome type 1 (LQT1) is caused by a mutation in *KCNQ1* (also known as *KVLQT1* or *K_v7.1_*), which encodes the pore-forming α subunits of the channels that generate *I_Ks_*, an adrenergic-sensitive slow outward potassium current. In 2010, Moretti *et al*. published a landmark study using PS-iPSCs generated from two patients with LQT1 with loss-of-function mutations in *KCNQ1* [119]. The iPSCs-cardiomyocytes derived from the LQT1 patients showed significantly prolonged action potential durations (APD) in ventricular and atrial subtypes as measured by whole-cell patch-clamp tests, and increased susceptibility to catecholamine-induced tachyarrhythmia. Additionally, there was a dominant negative trafficking defect associated with marked reduction in *I_Ks_*. Egashira *et al*. also generated iPSCs from a patient who had a novel mutation in *KCNQ1* and had survived ventricular fibrillation, in order to evaluate the pathological function of the mutation [120]. Using a multi-electrode array (MEA) system evaluating surface electrogenic activities of cell clusters, the authors demonstrated that there was a markedly prolonged field potential duration (FPD) in spontaneously beating EBs derived from the LQT1 patient’s iPSCs, which indicated a disturbance of repolarization. Since it is well known that *I_Ks_* and *I_Kr_* channels are involved in the repolarization process in a complementary manner, which is known as repolarization reserve [132], individual *I_Ks_* and *I_Kr_* channel blockers were administered to evaluate the functional states of each channel. It was found that the *I_Kr_* blocker, namely, E4031, significantly prolonged FPD, while the *I_Ks_* blocker, chromanol 293B, did not, suggesting that the *I_Ks_* channel was functionally affected by the novel gene mutation and that the repolarization was dependent on *I_Kr_*. This research demonstrates that PS-iPSC systems can be successfully applied in personalized diagnostics.

LQT syndrome type 2 (LQT2) is caused by a mutation in *KCNH2* (also known as *human ether-à-go-go*, *HERG*), which encodes the α subunits of channels that generate *I_Kr_*, a rapid component of the delayed rectifier potassium current. Itzhaki *et al*. demonstrated significant APD and FPD prolongation as well as arrhythmogenic findings, such as early afterdepolarization (EAD) and irregular beatings, in the LQT2-iPSC-cardiomyocytes. Additionally, those irregularities were successfully ameliorated by several drugs, including calcium-channel blockers, potassium (ATP)-channel openers, and late sodium channel blockers, which were evaluated using an LQT2-iPSC-cardiomyocyte screening system [121]. Matsa *et al*. also generated PS-iPSCs from two LQT2 patients: A daughter with arrhythmic events and her mother with the same mutation but without arrhythmia [122]. Intriguingly, despite a prolonged APD documented in both of the patients’ iPSC-cardiomyocytes, the daughter’s iPSC-cardiomyocytes exhibited stronger phenotypes. In the iPSC-cardiomyocytes from the daughter, an *I_Kr_* blocker and β-adrenergic agonist triggered EADs. Furthermore, the β-adrenergic antagonist nadolol and the potassium channel enhancer nicorandil shortened APD and abolished EADs. More recently, studies have revealed that genetic engineering strategies, such as targeted gene correction and mRNA knockdown by mutated-allele-specific RNA interference, rescued the electrophysiological phenotypes of the iPSC-cardiomyocytes from LQT2 patients [58,125]. These studies provide strong evidence that the mutated genes have an impact on the disease phenotypes.

LQT syndrome type3 (LQT3) is characterized by a prolonged QT interval resulting from persistent Na^+^ influx during repolarization due to a gain-of-function mutation in *SCN5A*, which encodes the α subunit of the cardiac Na^+^ channel. Terrenoire *et al*. generated PS-iPSCs from (a) a patient with a mutation in *SCN5A* and a polymorphism in *KCNH2*, a gene associated with LQT2; (b) from the patient’s mother who was homozygous for the same *KCNH2* polymorphism; and (c) from the patient’s father who was genetically normal [126]. An *in vitro* comparison between the iPSC-cardiomyocytes derived from these different individuals revealed that the *SCN5A* mutation, but not the *KCNH2* polymorphism, specifically contributed to the abnormality in Na^+^ channel kinetics. Ma *et al*. further demonstrated that mexiletine, a pure Na^+^ channel blocker, reversed the increased Na^+^ influx and prolonged APD [127].

LQT syndrome type 8 (LQT8), also known as Timothy syndrome (TS), is a rare disease caused by a single amino acid substitution in exon 8a of *CACNA1C*, the gene encoding for Ca_V_1.2 in humans. TS is multimodal disorder, including LQT syndrome, webbing of fingers and toes, immune deficiency, intermittent hypoglycemia, cognitive abnormalities, and autism [133]. Yazawa *et al*. reported that TS-iPSC-EBs contracted more slowly and irregularly, and ventricular-type iPSC-cardiomyocytes exhibited prolonged APD and had an increased incidence of delayed afterdepolarizations (DADs) [129]. Additionally, precise electrophysiological analyses, including Ca^2+^ imaging studies, revealed that excess Ca^2+^ influx and abnormal Ca^2+^ transients accompanied the aforementioned irregular electrical activities. It was further shown that roscovitine, a compound that increases the voltage-dependent inactivation of Ca_V_1.2, restored the electrical properties in the TS-iPSC-cardiomyocytes.

Catecholaminergic polymorphic ventricular tachycardia (CPVT) is an arrhythmogenic disorder characterized by stress-induced bidirectional or polymorphic ventricular tachycardia, and can lead to cardiac sudden death in children. CPVT is mostly caused by an autosomal dominant mutation in the cardiac ryanodine receptor (RYR) type 2 gene (*RYR2*) (CPVT type1), or more rarely an autosomal recessive mutation in the calsequestrin gene (*CASQ2*) (CPVT type2) [134,135]. These mutations result in an intracellular Ca^2+^ overload, which is released from the sarcoplasmic reticulum (SR), followed by arrhythmogenesis susceptible to sympathetic stimulations. Fatima *et al*. reported that CPVT1-iPSC-cardiomyocytes exhibited higher amplitudes and longer durations of spontaneous Ca^2+^ release events under basal condition [99]. Furthermore, after catecholaminergic stimulation, whole-cell patch-clamp tests on CPVT1-iPSC-cardiomyocytes showed frequent irregular beatings and putative DADs. Jung *et al.* reported that dantrolene, a drug for treating malignant hyperthermia by blocking skeletal RYR, restored aberrant Ca^2+^ sparks, abolished DADs, and triggered activity [100]. Itzhaki *et al*. further reported that flecainide and thapsigargin, specific inhibitors of the sarcoplasmic reticulum Ca^2+^ ATPase (SERCA2a) pump, restored abnormal electrophysiological properties [103]. Based on CPVT mouse-model experiments which showed that Ca^2+^/calmodulin-dependent serine-threonine protein kinase II (CaMKII) was related to arrhythmic events evoked by β-adrenergic stimulation, Pasquale *et al*. demonstrated that KN-93, an antiarrhythmic CaMKII inhibitor, also attenuated putative DADs and abnormal multifocal Ca^2+^ transients [105]. Given these results, CPVT1-iPSC-cardiomyocytes could be used to recapitulate clinical situations where arrhythmic events are caused by catecholaminergic stress and potentially provide insights into novel therapeutic strategies. Meanwhile, Novak *et al*. showed that β-adrenergic stimulation caused DADs and oscillatory arrhythmic prepotentials, which were accompanied by increases in resting intracellular Ca^2+^ in CPVT2-iPSC-cardiomyocytes [101].

### 5.2. Disease Modeling in Cardiomyopathy

Cardiomyopathies are generally categorized as idiopathic cardiomyopathies, including dilated cardiomyopathy (DCM), or hypertrophic cardiomyopathies (HCM), and are further categorized as specific cardiomyopathies, including several types of secondary cardiomyopathies resulting from ischemic, valvular, hypertensive, metabolic, and systemic diseases. Morphological observations and genetic information have been widely used to classify these cardiomyopathies, which could help to understand their pathophysiology [136,137]; however, only a few specific therapies are available for their treatment. To understand the underlying mechanisms associated with cardiomyopathies and to develop specific therapies, a relevant disease model is needed, for which cardiomyopathy-specific iPSCs have already been established. However, there are concerns about modeling cardiomyopathies with iPSCs because the clinical and genetic features of the patients with cardiomyopathies are highly variable, and thus far there have been no standard analytic methods to determine whether cardiomyocytes have a particular phenotype or not. We have reviewed recent reports while taking these points into consideration.

#### 5.2.1. Dilated Cardiomyopathy (DCM)

Genetic mutations are detected in approximately 20% of familial DCMs, which has led to the concept that DCM is a genetic disorder. DCM-iPSCs were first generated from a patient with a mutation in *LMNA* by Ho *et al*. [106]. *LMNA* encodes A-type lamin, a major component of the nuclear lamina that regulates nuclear structural integrity, chromatin organization, and telomere dynamics [138]. Although no clear nuclear phenotype was observed in the iPSCs from the DCM patient with the *LMNA* mutation, several cellular phenotypes, including decreased proliferation, increased cellular senescence, and augmented incidence of apoptosis by electrical stimulation, were observed. Siu *et al*. reported similar phenotypic results and demonstrated that a blockade of the extracellular signal-regulated kinase (ERK) pathway by mitogen activated protein kinase (MAPK)-ERK kinase 1 (MEK1) inhibitors attenuated the electrical stimulation-induced proapoptotic phenotypes on DCM-iPSC-cardiomyocytes [107]. Sun *et al*. generated iPSCs from family members with a mutation in *TNNT2* and from healthy family members without the mutation as controls [108]. The DCM-iPSC-cardiomyocytes often exhibited a disorganized layout of sarcomeric α-actinin, and the incidence of this sarcomeric disorganization was increased by norepinephrine stimulation. Additionally, Ca^2+^ imaging analyses demonstrated that the amplitudes of intracellular Ca^2+^ transients and Ca^2+^ storage in the SR were significantly smaller in the DCM patient derived iPSC-cardiomyocytes compared to those in controls. β-adrenergic blockers and overexpression of Serca2a improved the physiological functions of the DCM-iPSC-cardiomyocytes, which has been similarly observed in the failing myocardium of human and animals [139,140,141].

Systemic disease with hypertrophic cardiomyopathy. In 2009, a study by Carvajal-Vergara *et al*. reported the generation of PS-iPSCs from individuals with LEOPARD syndrome (LS), which was the first report on PS-iPSC research in cardiovascular diseases [118]. LS is caused by a missense mutation in *PTP11*, which encodes the protein tyrosine phosphatase SHP2, leading to dysregulation of RAS–MAPK signaling. Hypertrophic cardiomyopathy (HCM) is the most frequent cardiac anomaly observed in patients with LS, and is a potentially life-threatening problem in these patients. Protein samples extracted from LS-iPSCs were examined by phosphoproteomic microarray analysis to assess the molecular targets affected by *PTP11* mutation. This analysis revealed that there were significant differences in the phosphorylation levels of various proteins between LS-iPSCs and control iPSCs. In particular, the phosphorylation levels of ERK and MEK1 significantly differed under basal conditions, and under stimulation by basic fibroblast growth factor (bFGF), which induces the activation of the MAPK signaling pathway, indicating that LS-iPSCs could reproduce the RAS-MAPK signaling abnormality. Importantly, the cardiomyocytes from LS-iPSCs clearly exhibited cellular hypertrophy, and this was accompanied by a higher incidence of nuclear translocation of nuclear factor of activated T-cells (NFAT), which is an important regulator involved in cardiac hypertrophy. 

For other HCM-related diseases, PS-iPSCs have been established from individuals with Barth syndrome [59,113], carnitine palmitoyltransferase II deficiency [114], Friedreich’s ataxia [115,116,117], and Pompe disease [90]. Importantly, each of these studies showed, in part, some pathological phenotype in iPSCs and/or disease-relevant cells (see Table S1). As these disorders are extremely rare, the establishment of such PS-iPSCs could lead to the elucidation of the underlying pathologic mechanisms, and could further result in the development of specific treatments.

#### 5.2.2. Hypertrophic Cardiomyopathy (HCM)

As in the case of DCM, more than 1000 mutations in sarcomere-related genes, such as *MYH7* and *MYBPC3*, have been identified in 50%–60% of patients with HCM [142,143]. HCM is one of the most common type of cardiomyopathies with an estimated prevalence of 1:500 [144], and much research has been conducted regarding the molecular and physiological characteristics of HCM during the last two decades since the initial discovery of mutations in HCM patients [145]. HCM patient genetic analyses and animal HCM models have helped us understand the mechanisms of HCM. For example, we now know that sarcomeric mutations enhance calcium sensitivity and increase energy requirements [146]. However, therapeutic strategies still focus on the reduction of HCM-related symptoms as there are still no specific HCM therapies. Lan *et al*. first reported the generation of PS-iPSCs from five related HCM patients carrying a mutation in *MYH7* and five related healthy subjects without the mutation [110]. HCM-iPSCs-cardiomyocytes were multinucleated and hypertrophied with a higher incidence of NFAT nuclear translocation and upregulation of cardiac hypertrophy-related genes. Interestingly, irregular Ca^2+^ transients and arrhythmias had already occurred before hypertrophic change became obvious, suggesting that abnormal Ca^2+^ handling, including dysregulation of Ca^2+^ homeostasis and intracellular Ca^2+^ overload, could be upstream of the molecular mechanisms of HCM. It was also shown that the l-type Ca^2+^ channel blocker, verapamil, restored normal Ca^2+^ handling and rescued morphological and electrophysiological phenotypes in the HCM-iPSC-cardiomyocytes. Han *et al*. also used HCM-iPSC-cardiomyocytes to characterize HCM-related morphological phenotypes and electrophysiological abnormalities, including prolonged APD, arrhythmia, and abnormalities in multi-ion channels [111]. Interestingly, genome wide transcriptional profiling revealed that several genes, such as *WNT1* and *CDH1* associated with cell proliferation and cell motility, were upregulated in the HCM-iPSC-cardiomyocytes, indicating iPSCs could contribute to the understanding of the molecular mechanisms associated with HCM.

The onset of HCM often occurs during adolescence, but it remains unclear how HCM develops in patients with HCM-associated mutations, which suggests that there may be environmental factors that modify HCM disease progression. In other words, there may be interactions between genetic and environmental factors that contribute to HCM pathology [147]. To identify candidate environmental factors, our group generated HCM-iPSCs from three unrelated patients, and the cellular phenotypes of HCM-iPSC-cardiomyocytes were analyzed [112]. After stimulation of cardiomyocytes by several hypertrophy-promoting factors, it was found that endothelin (ET)-1 strongly induced HCM pathological phenotypes such as hypertrophy and intracellular myofibrillar disarray in the HCM-iPSC-cardiomyocytes. Additionally, a type A ET receptor antagonist, but not a type B antagonist, inhibited HCM phenotypes induced by ET-1. Although intercellular myofibrillar disarray is a well-recognized pathological hallmark of HCM, a functional role for intracellular myofibrillar disarray in HCM pathology remained unclear. Therefore, we then quantified the spontaneous contractile motion of HCM-iPSC-cardiomyocytes using a high-speed camera to capture the dynamic cellular motions. We found that cardiomyocytes stimulated by ET-1 showed contractile dispersion with randomly aligned myofibrils, suggesting that myofibrillar disarray is also a pathological marker, and contributes in part to cardiac dysfunction in HCM.

Arrhythmogenic right ventricular cardiomyopathy/dysplasia (ARVC/D). ARVC is characterized by fibrofatty replacement of the right ventricle myocardium, resulting in right ventricle chamber enlargement and dysfunction. Approximately 40%–50% of ARVC patients carry a mutation in one of several genes, most frequently in desmosome-related genes such as *PKP2*, which encodes plakophilin-2 [148,149]. Kim *et al*. established PS-iPSCs from two unrelated patients carrying different mutations in *PKP2* [96]. Under an artificial lipogenic milieu of insulin, dexamethasone, and 3-isobutyl-1-methilxanthine leading to the coactivation of peroxisome proliferator-activated receptor-α and proliferator-activated receptor-γ, lipogenesis and apoptosis were significantly induced in ARVC-iPSC-cardiomyocytes, further shifting fuel metabolism to glucose utilization similar to that observed in failing hearts. Ma *et al*. also generated ARVC-iPSCs carrying a mutation in *PKP2* and found that there was an accumulation of darker lipid droplets in ARVC-iPSC-cardiomyocytes [97]. Capsi *et al*. further demonstrated that pharmacological interventions could prevent lipid accumulation forced by adipogenic stress in ARVC-iPSC-cardiomyocytes [98].

#### 5.2.3. Other Models

The rapid increase in type 2 diabetes mellitus (T2DM) cases has considerably increased the clinical focus on diabetic cardiomyopathy [150,151]. Drawnel *et al*. demonstrated the conversion of control iPSCs to adult-type cardiomyocytes by manipulating metabolic substrates and also showed that structural and functional changes could be induced by the diabetic milieu of glucose, ET-1, and cortisol [53]. Additionally, the authors established PS-iPSCs from T2DM patient who developed cardiovascular disease (CVD) within five years of T2DM diagnosis and from patients who did not have cardiovascular diseases for more than 15 years after T2DM diagnosis. Interestingly, the iPSC-cardiomyocytes from T2DM patient who developed CVD within five years showed stronger cardiomyopathic phenotypes than T2DM patient with >15 years without CVD. Importantly, robust pharmacological screening was performed to determine candidate drugs that could rescue the cardiomypathic phenotypes using their iPSC-culture system.

In addition to the diseases in which cardiomyocytes are mainly affected, PS-iPSCs have been used to model non-cardiomyocyte-associated heart diseases such as Marfan syndrome [130] and supravalvular aortic stenosis [131]. In these non-cardiomyocyte analyses, efficient differentiation into the specific cardiovascular lineages and tissue-formation are also important.

## 6. Challenges in Cardiovascular Disease Modeling

**Figure 2:**
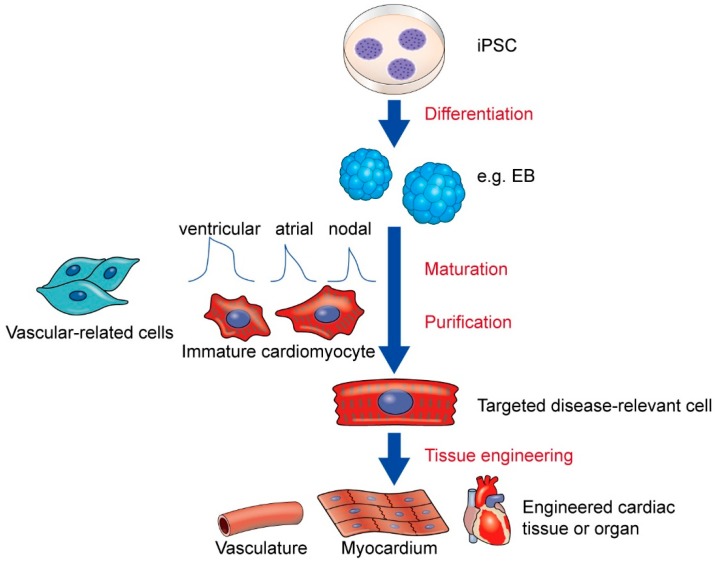
Steps toward clinical application and disease modeling using iPSCs. iPSCs can be differentiated into cells from all three germ layers via several methods, including via EB forming protocols. While cardiomyocytes can be isolated from spontaneously formed EBs, their cellular characteristics such as electric subtype (ventricular, atrial, or nodal type) and/or maturity are heterogeneous. Additionally, EBs potentially contain various types of non-cardiovascular cells, including endothelial and vascular smooth muscle cells. In such heterogeneous differentiated cell populations, site-specific propensities of coronary, pulmonary, or cerebral blood cells would also be uncertain. Therefore, to model organ- or site-specific diseases, a more specific approach for cellular differentiation is needed. Thus, stepwise strategies should be considered to obtain ideal disease-relevant cells. Tissue engineering techniques could help build cardiovascular systems, leading to comprehensive disease modeling to further enhance drug monitoring and the development of replacement therapies in the future. EB, embryoid body; iPSC, induced pluripotent stem cell.

While it has been six years since PS-iPSC-cardiomyocytes have been successfully generated and used to model pathophysiological phenotypes *in vitro*, this system still has methodological and experimental hurdles to overcome. To make steady progress toward clinical applications, multi-stepwise technical advances are needed (Figure 2). First, the most important issue is that cardiomyocytes derived from iPSCs are different from physiological adult cardiomyocytes *in vivo*. Indeed, many researchers have shown that iPSC-cardiomyocytes are not functionally identical to adult-type cardiomyocytes; rather, they show immature cellular phenotypes [152,153,154,155,156]. Ultrastructural studies have also revealed immature sarcomeric structures without T-tubules in ESC- and iPSC-cardiomyocytes [157,158]. To convert the iPSC-cardiomyocytes into mature phenotypes, various techniques have been employed, such as long-term culture [159,160,161], increased substrate stiffness [162], electric stimulation [163], and biochemical arrangement [164,165,166]. Furthermore, because of the differences in metabolic substrates between fetal and adult cardiomyocytes, recent studies have promoted cardiomyocyte maturation through metabolic and propathogenic manipulation in culture conditions. This technique may potentially enable iPSCs to model adult onset and environmental factor-dependent diseases with metabolic phenotypes [53,96].

Second, the purity of cardiomyocytes during the differentiation process is of great importance. The population of differentiated iPSCs is heterogeneous and contains many types of cells, including those that are still undifferentiated. In addition, there are at least three subtypes of cardiomyocytes (ventricular, atrial, and nodal), which can be distinguished according to electrophysiological properties and gene expression patterns at the single cell level [44,167]. It is therefore expected that heterogeneous populations of cardiomyocytes in experiments will induce great variance and errors. Furthermore, large-scale purification of cardiomyocyte populations to ensure the absence of undifferentiated cells is essential in replacement therapies for cardiac tissue repair. Many groups have also tried to develop directional methods to differentiation ESCs and iPSCs into specific subtypes by regulating molecular pathways with defined media [168,169,170,171,172]. Cardiomyocyte-purification methods that utilize cardiac-specific metabolic characters and cardiomyocyte-specific markers have also been reported [173,174].

Cardiac tissue is composed of highly organized cell populations, such as myofilaments from individual cardiomyocytes, endothelial cells, vascular smooth muscle cells, interstitial fibroblasts, and extracellular matrix components. Since cardiovascular diseases can often arise from the dysfunction of cells that are not always cardiomyocytes, and from multicellular dysfunctions between cardiomyocytes and non-cardiomyocytes, cardiomyocyte-specific analyses cannot model all cardiac diseases. For this reason, it would be best if cardiovascular diseases could be modeled using a combination of various cell types, including cardiomyocytes, fibroblasts, endothelial cells, and vascular smooth muscle cells, which can all be differentiated from iPSCs [175,176,177]. Technical advancements that focus on the cellular interactions and interconnections between cardiomyocytes and non-myocytes within three-dimensional tissue structures are thus required [178]. Eschenhagen and other researchers have reported the utility of hydrogel-based engineered heart tissue (EHT) technology using cardiomyocytes from animal models and iPSCs [179,180,181,182]. As one of its advantages, EHT from neonatal rats exhibited mature cardiomyocyte phenotypes as demonstrated by their morphological and electrophysiological properties [183]. This technology was successfully used to reproduce these adult phenotypes in *Mybpc3*-targeted knock-in (HCM-model) mice [184]. Recently, Nunes *et al*. created a new platform for EHT, dubbed “biowires,” which is a three-dimensional cardiac tissue generated from the self-assembly of ESC- and iPSC-cardiomyocytes and supporting cells in combination with geometric electrical stimulation [185]. The biowires induced highly organized cardiac architecture and mature cellular phenotypes. Moreover, co-cultures of three-dimensionally aligned cardiomyocytes with non-cardiomyocytes, such as endothelial cells, promoted cardiac function and prolonged cell survival in grafts, showing the importance of multi-cellular interactions within cardiac tissues [186,187]. Taken together, these results indicate that it could soon be technically possible to perform drug screening and disease modeling in three-dimensional and well-allocated cardiac tissues using PS-iPSC systems, which could better reflect physiological conditions *in vivo*.

## 7. Summary and Future Perspectives

Generation of iPSC systems has given rise to a great paradigm shift in biomedical research. Based on the accumulated molecular and functional analyses in conventional ESC systems, iPSC systems have rapidly developed into a valuable platform for regenerative medicine, disease modeling, and drug development. Academic-industrial alliances between medical doctors, biomedical researchers, and pharmaceutical companies will become increasingly important as iPSC systems move forward toward clinical use. While there are still some missing pieces of the puzzle in iPSC systems, we are confident that those hurdles can be overcome in the near future, leading to the development of novel medical fields through the use of versatile iPSCs.

## Supplementary Materials

Supplementary materials can be found at http://www.mdpi.com/1422-0067/16/08/18894/s1.
